# Indoor environment dataset based on RSSI collected with bluetooth devices

**DOI:** 10.1016/j.dib.2024.110692

**Published:** 2024-06-28

**Authors:** Yuri Assayag, Horacio Oliveira, Max Lima, João Junior, Mateus Preste, Leonardo Guimarães, Eduardo Souto

**Affiliations:** Institute of Computing, Federal University of Amazonas, Amazonas, Brazil

**Keywords:** Indoor localization, Bluetooth, Received signal strength indicator, Internet of things, Location based services

## Abstract

This paper describes a data collection experiment focused on researching indoor positioning systems using Bluetooth Low Energy (BLE) devices. The study was conducted in a real-world scenario with 150 test points and collected signals from 11 mobile devices. The dataset contains RSSI values from the mobile devices in relation to 15 fixed anchor nodes in the experimentation scenario. The dataset includes data on device identification, labels and coordinates of test points, and the room where the data was collected. The data is organized as CSV files and offers valuable information for researchers developing and assessing location models. By sharing this dataset, we aim to support the creation of robust and precise indoor localization models.

Specifications Table

**Table:** 

Subject	Computer Science.
Specific subject area	Indoor Localization; Internet of Things.
Type of data	Each device is an individual .CSV file.
Data collection	We placed 150 test points in the scenario, about 2 m apart, to gather RSSI samples. Using 11 mobile devices, we collected signals at all test points. The devices sent Bluetooth advertising packets, which were received by several anchor nodes. The anchors calculated the RSSI of the packets and sent the data to a central device for storage. Our collection app marked the current device position on a map to accurately record the real position of each test point, ensuring precise data collection.
Data source location	City/Town/Region: Manaus Country: Brazil GPS Coordinates: -3.088334, -59.964559
Data accessibility	Data hosted in public repository. Repository name: Mendeley Data. DOI: 10.17632/3v9mcsvcd2.1 Direct URL to data: https://data.mendeley.com/datasets/3v9mcsvcd2/1
Related research article	Y. Assayag, H. Oliveira, E. Souto, R. Barreto and R. Pazzi, “Adaptive Path Loss Model for BLE Indoor Positioning System”, in IEEE Internet of Things Journal, vol. 10, no. 14, pp. 12898-12907, July 15, 2023, doi: 10.1109/JIOT.2023.3253660. [1]

## 1 Value of the Data

• This dataset includes information on signal strength collected from Bluetooth devices in a real indoor environment. Each sample contains RSSI values collected from a mobile device in relation to 15 different anchor nodes (also known as access points or gateways) within the environment.
• The data was gathered in a school environment, including multiple classrooms and halls, enabling the identification of mobile device locations at the classroom level and the precise region within the room.
• The dataset consists of 15.000 samples from 11 mobile devices across 150 test points within the scenario. This data provides a solid foundation for further research in indoor localization.
• The data allows researchers to test new localization algorithms and assess the accuracy of proposed models or average error per test point since each sample includes the specific data collection location. It supports both model-based and fingerprint-based positioning systems.
• Researchers can use the dataset to study RSSI behavior across various devices and conditions and compare these findings with different signal propagation models. This can lead to a better understanding of wireless communication efficiency.
• Access to this real-world sensor data benefits research centers and the academic community, especially those without the resources or time to create their own datasets.

## 2 Background

The dataset was developed to assess and test novel methods for indoor localization systems that use RSSI (Received Signal Strength Indicator) to locate mobile devices in indoor environments. Traditional methods [2,3] often depend solely on RSSI data from a mobile device in relation to a few stationary anchor nodes within limited experimental settings. Additionally, existing databases [[4], [5], [6], [7]] primarily focus on fingerprinting solutions that use only RSSI values and test point identifications. Although beneficial, these datasets lack the coordinates of the locations where fixed nodes were installed, which is vital information for evaluating methods based on signal propagation models.

Data were gathered using specialized commercial devices designed for this purpose, employing Bluetooth technology for communication between devices. This approach offers a significant advancement in localization using Bluetooth Low Energy devices, bypassing the need for WiFi. This article is connected to a research paper on localization by Assayag et al. [1], where we introduce a novel localization approach based on a signal propagation model and compare it to other widely used techniques in existing literature.

Overall, our dataset enables the development and assessment of new indoor localization strategies and allows for an in-depth examination of RSSI behavior across various mobile devices in real-world experimental scenarios.

## 3 Data Description

This dataset aims at enhancing indoor localization research by offering various CSV files containing signal strength data from devices equipped with Bluetooth Low Energy. The data was gathered at 150 test points within an actual school setting with several classrooms. Only our team was present during data gathering. Each CSV file represents signal strength measurements recorded by a distinct mobile device across all areas of the experimental setup. The data collection process used a sampling rate of 1 s, facilitating optimal tracking for localization systems.

The CSV files are structured according to the unique identification codes assigned to the mobile devices used during data collection. The identifications of different devices within the provided dataset are: 1F61, 10B2, 10CE, 20B5, 104D, 2055, 121B, 121D, 1210, 1211, 1212, and 2055. Each mobile device has a corresponding .csv file, for example 1F61.csv.

**Fig. 1:**
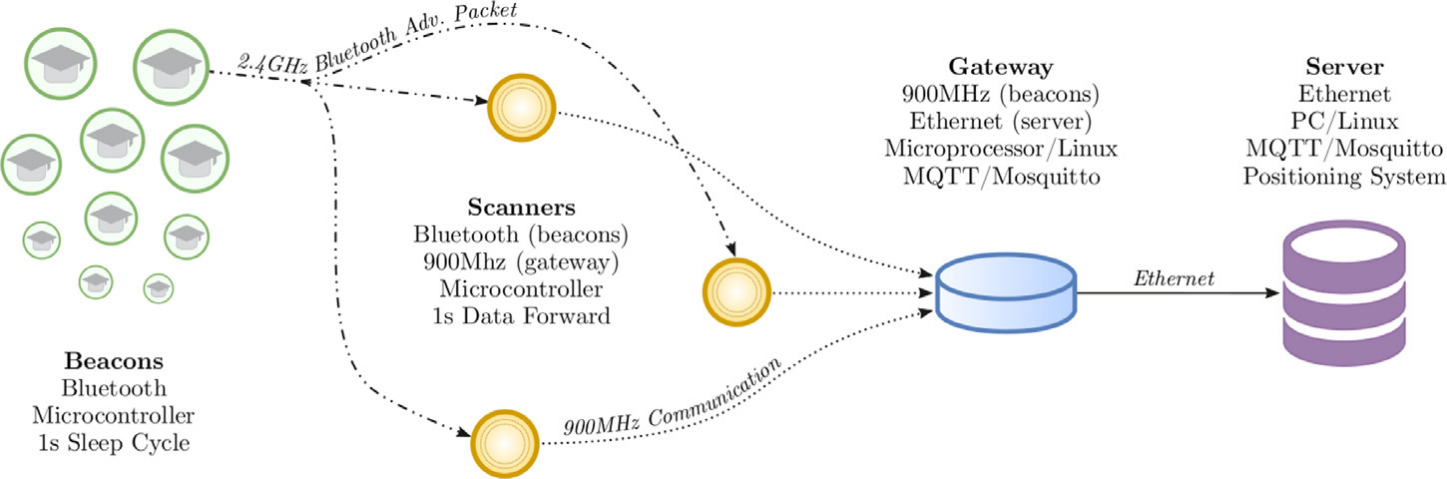
Experimentation scenario composed of 15 anchors (in blue) fixed to the ceiling of the rooms and 150 RPs (in gray dots) with a spacing of about 2 m in the rooms. (For interpretation of the references to color in this figure legend, the reader is referred to the web version of this article.)

The file structure has the following columns: WAP#ID, LABEL, X, Y, DEVICE, and ROOM_ID. Detailed information about each of the variables is found in Table 1. The values in columns X and Y are based on the origin point of the environmental map, as shown in Fig. 1. The label data can serve as input for machine learning models to predict the location of a mobile device, while the (X, Y) data enables the calculation of the average error should the learning model estimate a test point different from the true one. By utilizing the LABEL, X, and Y columns, one can assess the accuracy and average error of a model.

**Table 1:** A description of the columns of each of the datasets.

Column	Type	Meaning	Example
WAP-#ID	Numeric (dBm)	These columns include values for the Received Signal Strength Indicators (RSSI) measured in dBm. These measurements are estimated by the mobile device in relation to each of the two sets of 15 WAPS (Wireless Anchor Points) placed throughout the experimental environment.	-87
LABEL	String	This column contains labels for the test points where signal intensities were collected. There are 150 distinct test points included in the dataset.	16_1_1
X	Numeric	This column shows the values of the 2D horizontal coordinates of the test point where the sample was taken.	6.1
Y	Numeric	This column shows the values of the 2D vertical coordinates of the test point where the sample was taken.	10.5
DEVICE	String	This column contains the identification code of the mobile device used to gather the data.	104D
ROOM_ID	String	This column contains the identification of the room or hall where the data was collected.	ROOM_16

Besides the mentioned datasets, the folder also includes the ANCHORS.csv file, which provides the identification of each of the 15 anchors along with their location coordinates (X, Y) within the environment. This information is valuable for computing the distance between the test points and each of the two anchors, particularly for solutions based on propagation models.

## 4 Experimental Design, Materials and Methods

This paper proposes a new indoor localization dataset, a Bluetooth-based solution that allows locating the position of people inside a school. On the hardware side, we present a complete architecture that uses Bluetooth beacons (known as mobile device) to send packets to anchor nodes that estimate the received signal strengths (RSSIs) and send them directly to the gateway. The beacon nodes are both low-priced and energy-efficient.

Our data are all based on a real-world, large-scale implementation of our proposed IPS in a school building composed of different rooms in a total area of about 720 m². Fig. 1 shows an overview of the floor plan of the school in which we implemented the testbed as well as the location of the anchor nodes. Given the size of our scenario and the time required to collect data throughout the environment, we choose to maintain a 2 m spacing between test points within each room. This spacing allows us to gather representative data from various regions of the room. To define test points, we start by measuring 0.5 m from the edge between two walls using a tape measure, setting this as the starting point. From there, we measure 2 m from the starting point to establish subsequent points until all points in the room are covered. Table 2 shows the number of test points in each room.

**Table 2:** A description of the room sizes and the number of test points in each room.

Room ID	Width (m)	Height (m)	Test Points (TP)
Room 1	5.7	8.7	12
Room 2	3.5	7.2	8
Room 3	6.0	6.8	9
Room 4	8.0	6.8	12
Room 5	6.8	6.8	9
Room 6	8.2	6.8	12
Room 7	6.8	8.5	12
Room 8	8.0	6.5	12
Room 9	8.0	6.5	12
Room 10	8.0	6.5	12
Room 11	10.0	6.5	15
Hall 1	3.0	6.5	8
Hall 2	15.0	2.0	7
Hall 3	16.0	2.0	8

We trained most of the school area with 150 different test points. For each test point, we sent packets from 11 mobile devices, where all devices are equipped with similar hardware located in different parts of the users’ body such as the arm, pockets, and backpack, resulting in 15.000 samples in the dataset.

The equipment used, manufactured by our Positivo Technology team, employs Bluetooth Low Energy (BLE) technology for communication. All 11 beacons (or mobile devices) are from the same manufacturer and feature identical systems, models, and antennas. The system operates simply by periodically sending BLE advertising packages. We chose devices with uniform hardware to ensure that all users of the location system receive a bracelet with the same hardware, enabling user location within the school environment without any user intervention.

In our scenario, we discarded the use of WiFi technology, since they tend to consume more energy and, thus, we decided to use the Bluetooth BLE technology. Furthermore, one of the premises of the proposed architecture was to not rely on the WiFi infrastructure of the building where it was going to be installed. To ensure optimal coverage, our aim is to maintain at least one anchor node by room. However, in certain scenarios, placing anchor nodes in the ideal locations can be challenging, whether due to accessibility issues or aesthetic considerations, such as in shopping malls. In our specific case, operating within a school environment, we encountered restrictions regarding access to electrical wiring for anchor node installation. Consequently, we positioned the anchor nodes near points of easy access to the electrical infrastructure, which, in all rooms, happened to be on the ceiling. As a result, with the exception of anchor nodes 13, 14, and 15, which were mounted on hall walls at a height of 2.5 m, all other anchor nodes were affixed to the ceilings of the rooms at a height of 3.0 m.

The rooms vary in size, leading to a different number of test points in each room, ranging from 8 to 15. Table 2 shows the size of each room along with the corresponding number of test points. In many studies concerning indoor location systems, authors often test their proposals in small-scale scenarios, where there are minimal or no obstacles between the transmitting device and the receivers. In contrast, we believe that a realistic experimental setup should incorporate environmental elements that introduce obstacles between devices, such as walls. Hence, our experimental scenario comprises different environments of varying sizes. All rooms and halls are partitioned by walls, which directly affect the signal power values, mirroring behaviors observed in numerous real-world scenarios.

In the Bluetooth specification, an advertiser is a “BLE device that broadcasts advertising packets during advertising events on advertising channels”. An advertising event is a series of three advertising packets on different advertising physical channels (37, 38, and 39) sent by the advertiser.

In our architecture, the mobile devices are the BLE advertisers. They achieve the lowest possible power consumption by simply waking up, triggering an advertising event, and going back to sleep. An anchor node is a BLE device that listens for advertising events on the physical advertising channels. Since the anchor can only listen to a single channel at a time, it starts listening to the first advertising channel (37) for a time and, then, changes to the next channel, and so on. Even though the Bluetooth specification requires the anchor node to report the RSSI of the received advertising packets, it does not require nor suggest how to report the channel in which the packet was received.

**Fig. 2:**
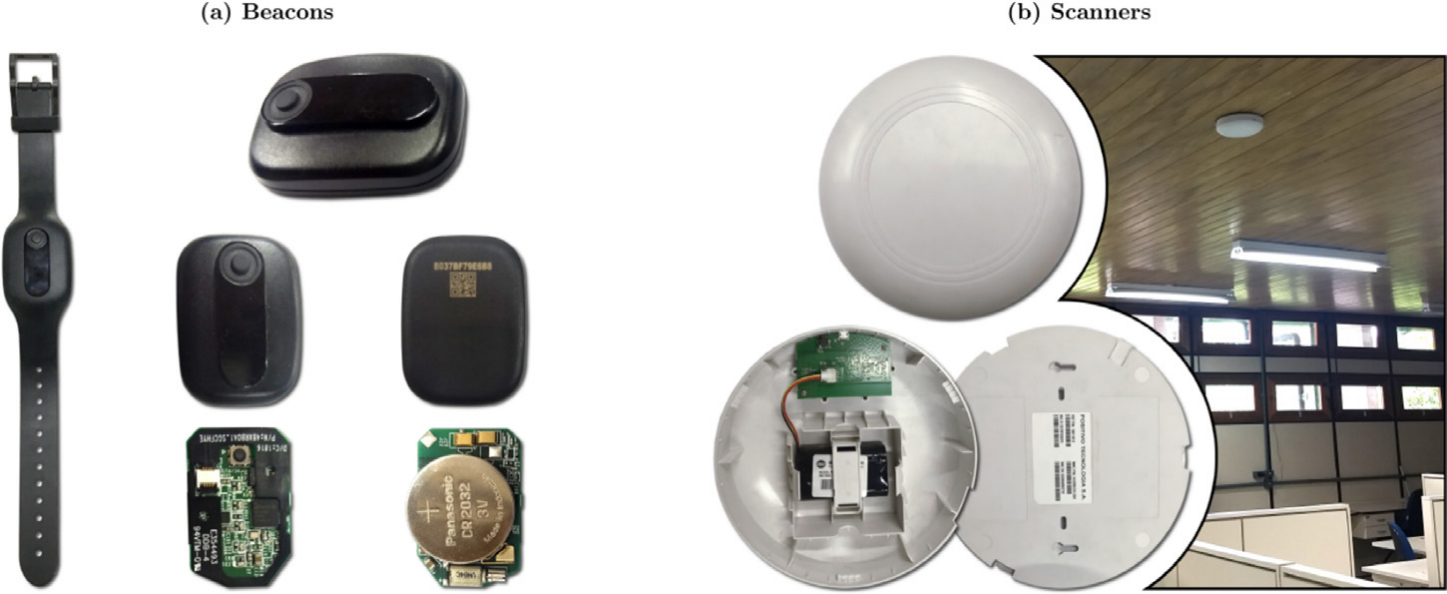
All beacons send a Bluetooth advertising packet. These packets are received by the anchor nodes that estimate and forward the RSSI data to the gateway using a 900 MHz, long-range, communication. The gateway sends the data to the server that is capable of generating the dataset.

The IPS architecture is depicted in Fig. 2. In this architecture, mobile devices send Bluetooth advertising packets every 100 ms. However, as previously mentioned, obstacles have a significant impact on wireless signal behavior. Additionally, the indoor environment's characteristics result in multiple signal paths, causing RSSI variation. Hence, we opted not to utilize raw RSSI data in our database. Each sample in our database corresponds to 1 s of data collection from a mobile device, whereas the device transmits advertising packets every 100 ms. Consequently, within 1 s of collection, certain RSSI values may exhibit peaks (or outliers), potentially introducing noise into the database. To mitigate this issue, we implemented a simple filter that only keeps the average RSSI for 1 s of collection, forming the final sample stored in the database. In essence, each line in the database represents the average RSSI of 1 s of collection. This approach is commonplace and widely adopted in the literature.

**Fig. 3:**
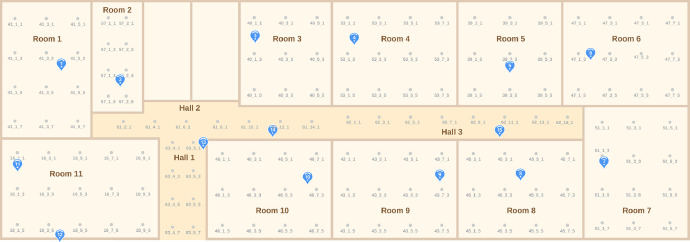
(a) Beacons with Bluetooth communication; one beacon is inside a bracelet, as worn by the users; the other beacons are outside the bracelet (front and back), and opened to show hardware (front and back); and (b) scanners with Bluetooth and 900 Mhz communication (front, opened, back, and installed on the ceiling). The gateway has a similar hardware footprint as of the anchor nodes.

The anchor nodes compute the RSSI of the received packets and send them directly to a central device, the gateway, using long-range, 900 Mhz communication. The gateway is connected to a server, which brings together all signal information in the database. The hardware is depicted in Fig. 3 except for the gateway, which has the same footprint as the anchor, and for the server, which is a standard PC (Intel i7, 16GB of memory).

In our database, each row, referred to as a sample, represents the RSSI data collected every second by a mobile device. With 150 distinct test points and 100 data samples collected per point, each point requires approximately 1.5 min for signal collection. This sampling rate was selected because 100 samples per point provide sufficient data for a machine learning model to differentiate signal characteristics across different regions of the scenario. Moreover, it helps mitigate the tedium and time constraints associated with data collection, especially considering the scale of our experimental environment.

## Limitations

The study could be improved by exploring two potential areas for expansion. First, gathering data throughout different months of the year and their impact on the RSS values detected by mobile devices. Second, testing a larger number of classrooms could increase the volume of data collected.

## Ethics Statement

The authors have read and followed the ethical requirements for publication in Data in Brief. The devices were implemented in a school environment, however, access to the area was limited to only the authors involved in this article. Therefore the work does not involve others outside of this project and no personally identifiable information was collected as part of the data collection process.

## CRediT Author Statement

**Yuri Assayag**: Manuscript Authoring, Lab Setup, Data Collection, Data Analysis; **Horácio Oliveira**: Manuscript Authoring, Lab Setup, Writing-Reviewing and Editing; **Eduardo Souto**: Manuscript Authoring, Writing-Reviewing and Editing; **Max Lima**: Lab Setup, Data Collection, Data Analysis; **Joao Junior**: Data Collection, Data Analysis; **Mateus Preste**: Data Collection, Testbed implementation; **Leonardo Guimarães**: Data Analysis, Testbed Implementation.

## Data Availability

Indoor Environment Dataset Based on RSSI Collected with Bluetooth Devices (Original data) (Mendeley Data).
